# Expression and Localization of Lung Surfactant Proteins in Human Testis

**DOI:** 10.1371/journal.pone.0143058

**Published:** 2015-11-24

**Authors:** Stephanie Beileke, Horst Claassen, Walter Wagner, Cord Matthies, Christian Ruf, Arndt Hartmann, Fabian Garreis, Friedrich Paulsen, Martin Schicht, Lars Bräuer

**Affiliations:** 1 Institute of Anatomy II, Friedrich-Alexander-University Erlangen-Nürnberg, Erlangen, Germany; 2 Institute of Anatomy and Cell Biology, Martin-Luther-University Halle-Wittenberg, Halle (Saale), Germany; 3 Federal Armed Forces Hospital Hamburg, Department of Urology, Hamburg, Germany; 4 Institute of Pathology, University Hospital Erlangen, Erlangen, Germany; University of Nevada School of Medicine, UNITED STATES

## Abstract

**Background:**

Surfactant proteins (SPs) have been described in various tissues and fluids including tissues of the nasolacrimal apparatus, airways and digestive tract. Human testis have a glandular function as a part of the reproductive and the endocrine system, but no data are available on SPs in human testis and prostate under healthy and pathologic conditions.

**Objective:**

The aim of the study was the detection and characterization of the surfactant proteins A, B, C and D (SP-A, SP-B, SP-C, SP-D) in human testis. Additionally tissue samples affected by testicular cancer were investigated.

**Results:**

Surfactant proteins A, B, C and D were detected using RT-PCR in healthy testis. By means of Western blot analysis, these SPs were detected at the protein level in normal testis, seminoma and seminal fluid, but not in spermatozoa. Expression of SPs was weaker in seminoma compared to normal testicular tissue. SPs were localized in combination with vimentin immunohistochemically in cells of Sertoli and Leydig.

**Conclusion:**

Surfactant proteins seem to be inherent part of the human testis. By means of physicochemical properties the proteins appear to play a role during immunological and rheological process of the testicular tissue. The presence of SP-B and SP-C in cells of Sertoli correlates with their function of fluid secretion and may support transportation of spermatozoa. In seminoma the expression of all SP's was generally weaker compared to normal germ cells. This could lead to a reduction of immunomodulatory and rheology processes in the germ cell tumor.

## Introduction

Surfactant proteins (SPs) were first detected in the human lung [1,2]. The proteins differ considerably in structure, function and biochemical properties. SP-A and SP-D are representatives of the C-type lectins that have immunological functions in non-specific and specific immune defense [3–5]; SP-B and SP-C are among the smallest and most hydrophobic proteins of all. Their physicochemical properties enable them to reduce the surface tension of biological interfaces and contribute to the adsorption of phospholipids at the air-liquid interface [6,7].

SP-A and SP-D, both belong to the C-type lectin family—in the C-type lectin mechanism, the proteins bind to specific carbohydrates of bacteria, protozoans, fungi, and viruses [8,9]. This is followed by opsonization and accelerated immune defense reactions to these microorganisms [3–5]. SP-A and SP-D were detected in various tissues including human nasal mucosa, digestive tract, tear ducts, salivary glands of the head and gingiva [10–14]. By contrast, SP-B and SP-C feature very low molecular weights and hydrophobic proteins. They are important for the formation of surfactant monolayers. Due to their lower surface tension they can stabilize air-fluid interfaces [6,7,15]. The presence of SP-B and SP-C has been reported in a variety of tissues and fluids, including tissues of the nasolacrimal apparatus and eye surface, in tear fluid, in salivary glands, in the gingiva, and in saliva [11,13,16].

The human testis, as the male gonad, is an endocrine as well as exocrine gland. Testicular tissue thus comprises mainly two types of functional tissue. The exocrine function, in particular the reproductive system, is based on germ cells, i.e. spermatogenesis including development of spermatogonia and maturation to spermatozoa. The stromal cells include cells of Leydig for endocrine testosterone production and cells of Sertoli for germ cell support.

Malignant tumors of the testis are the most common tumor in men under the age of 40 with an average incidence of 9/100,000/year in Europe. Germ cell tumors represent 90% of testicular tumors. They are classified as seminoma (55%) or non-seminomatous germ cell tumors like teratomas, embryonal carcinoma or yolk sac tumors. 45% of germ cell tumors are composed of both seminomatous and non-seminomatous components (mixed germ cell tumours) [17]. The cure rate is close to 100% in early stages and about 50% in advanced, poor-prognosis stages [18]. A knowledge of molecular markers is of special interest for tumor classification and staging.

No proof of expression of SPs has been found to date in the healthy testis. In this study, we confirmed the presence of surfactant proteins A, B, C and D for the first time. We also characterized presumed surface regulatory, immunological and rheological properties required for the testicular ducts to function and for cells of Sertoli and Leydig. The presence of surfactant proteins within testicular tissue is, moreover, related to immunomodulatory and antimicrobial functions. Furthermore, we characterized the significance of these SPs within the framework of testicular cancer.

## Materials and Methods

### Sampling of human tissue

Tissue samples from patients with seminoma comprised 10 men in the age of 20, 25, 26, 32, 38, 40, 44, 46, 49, and once again 49 years. Tissue sample from patients with teratoma comprised 3 men in the age of 21, 27, and 31years of age. All patients were informed and written consent for excision and scientific use of testis tissue were obtained. Methods included proper consent and approval, complied with the declaration of Helsinki. The study reviewed and approved by the Ethics Committee of the Medical Association of Hamburg (MC-227/10) is entitled "Detection of gene expression changes in testicular tumors". The pathological samples are obtained from surgical-material and were stored at -20°C.

The tissue samples of human lung, normal testis and prostate were obtained from cadavers (5 male, 11 female, aged 33–76 years) donated to the Department of Anatomy and Cell Biology, Martin-Luther-University Halle-Wittenberg, Germany. Experiments on human tissue were done in accordance with the provisions of the Declaration of Helsinki for research involving human tissue. Prior to death every body donor dedicated his body to the Institute for Anatomy and Cell Biology for scientific and academic purposes, stating this in his last will.

The used samples were dissected from the cadavers within a time-frame of 12–24 h postmortem. Previous to dissection, the history of each cadaver was studied. Samples that were affected by acute infections, tumors, recent traumata or surgical operations were not used in this study. Furthermore, all samples with a post-mortal interval longer than 24 hours were omitted. After resection, a part of the specimens were fixed in 4% paraformaldehyde for later paraffin embedding; the other half of the specimens were used for molecular biological investigations and thus immediately frozen at -80°C. The investigated semen samples were donated by fertile volunteers after approval of ethics and informed consent. Tissue and semen sample were after adding Triton-buffer (300μl), protease- and phosphatase-inhibitor (10μl/1ml each) homogenized on ice with an Ultra-Turrax T25 homogenizer (Janke & Kunkel, Staufen i. Br., Germany). Incubation for 30 min on ice, centrifugation and another incubation for 20–30 min at 4°C followed. After centrifugation for 30 min at 16,000 x g, the supernatant was transferred to reaction tubes. The total protein content was measured using Bradford reagent, BIO-RAD Protein Assay (Bio-RAD, Munich, Germany).

### RNA preparation and complementary DNA synthesis (cDNA)

For the reverse transcriptase polymerase chain reaction (RT-PCR), tissue biopsies from testis and seminoma samples were crushed in an agate mortar under liquid nitrogen, then homogenized in 5 ml peqgold RNA pure solution (peqLab Biotechnologie, Erlangen, Germany) with a Polytron homogenizer. The collected samples were treated under the same conditions except for crushing under liquid nitrogen. Insoluble material was removed by centrifugation 12,000g, 5 min, 4°C). Total RNA was isolated using RNeasy-Kit (Qiagen, Hilden, Germany). Crude RNA was purified with isopropanol and repeated ethanol precipitation and contaminating DNA was destroyed by digestion with RNase-free DNase I (20 min 25°C; Boehringer, Mannheim, Germany). Contamination of the purified RNA by genomic DNA was prevented by performing PCR with specific primers for SPs as well as for ß-actin. In no case was amplification obtainable using the purified RNA as a template. The DNase was heat-denatured for 15 min at 65°C. 500 ng RNA were used for each reaction: cDNA was generated with 50 ng/μl (20 pmol) oligo (dT)15 primer (Amersham Pharmacia Biotech, Uppsala, Sweden) and 0.8 μl superscript RNase H reverse transcriptase (100 U; Gibco, Paisley, UK) for 60 min at 37°C. The ubiquitously expressed ß-actin, which proved amplifiable in each case with the specific primer pair, served as the internal control for the integrity of the translated cDNA.

### Polymerase Chain Reaction (PCR)

For conventional PCR, 2 μl cDNA (from each sample) was incubated with 13.7 μl H_2_O, 1 μl 50 mM MgCl_2_, 0.5 μl dNTPs, 2 μl 10x PCR buffer, 0.2 μl (5 U) Taq DNA polymerase (Invitrogen) and 0.5 μl (100 pmol) of each of the following primers: SP-A sense 5’-GAT GGG CAG TGG AAT GAC AGG-3’, antisense 5’-GGG AAT GAA GTG GCT AAG GGT G-3’ (212 bp); SP-B sense 5’-CAA ACG GCA TCT GTA TGC AC-3’, antisense 5’-CGG AGA GAT CCT GTG TGT GA-3’ (194 bp); SP-C sense 5’-TCA TCG TGG TGA TGG TG-3’, antisense 5’- ATG GAG AAG GTG GCA GTG GTA A-3’ (110 bp); SP-D sense 5’-ATG TTG CTT CTC TGA GG-3’, antisense 5’-TCA GAA CTC GCA GAC CAC AAG-3’ (461bp). For the negative control water was used instead cDNA. After 5 min of heat denaturation at 96°C, the PCR cycle consisted of (1) 96°C for 60 s, (2) 57°C (SP-A, SP-B, SP-C, and SP-D) for 60 s each, (3) 72°C for 60 s. 35 cycles were performed with each primer pair. The final elongation cycle consisted of 72°C for 4 min. The primers were synthesized by MWG-Biotech AG, Ebersberg, Germany. 10 μl of the PCR product was loaded onto agarose gel and after electrophoresis the amplified products were visualized using fluorescence. Bp values were compared with gene bank data [19]. For verification and comparison, bacterial plasmids carrying the genes for the investigated proteins were used as a reference and positive control (German Resource Centre for Genome Research GmbH; SP-A: IRAUp969H0686D6; SP- B: IRAKp961K1368Q2, SP-C: IKAUp969F0244D6, SP-D: IRAUp969D0386D6). PCR products were also confirmed by BigDye sequencing (Applied Biosystems, Foster City, CA). To estimate the amount of amplified PCR product, we performed a ß-actin PCR with specific primers (sense 5’- CAA GAG ATG GCC ACG GCT GCT-3’, antisense 5’- TCC TTC TGC ATC CTG TCG GCA-3’, 275 bp) for each investigated tissue. We applied the above-mentioned conditions for this additional PCR. 10 μl of the PCR product was loaded onto the agarose gel. The base pair marker (Mass Ruler DNA-Ladder, Fermentas, St. Leon-Rot, Germany) used for each PCR ranges from 80 to 1031 bp.

### Quantitative Real-Time RT-PCR (qRT-PCR)

Gene expression was analyzed with a LightCyler480 (Roche Holding, Mannheim, Germany). Real-time RT-PCR was performed using the following primers: SP-A sense 5’-CTG TCC CAA GGA ATC CAG AG-3’, antisense 5’- CCG TCT GAG TAG CGG AAG TC-3’ (120 bp); SP-B sense 5’-GAT CAA GCG GAT CCA AGC CAT-3’, antisense 5’-AGC AGG ATG ACG CAG TAG CGC T-3’ (110 bp); SP-C sense 5’-CTG GTT ACC ACT GCC ACC TT-3’, antisense 5’-TCA AGA CTG GGG ATG CTC TC-3’ (142 bp); SP-D sense 5’-TGC TGC TCT TCC TCC TCT CTG C-3’, antisense 5’-GGG CGT TGT TCT GTG GGA GTA G-3’ (95bp). The PCR reaction contained 10 μL LightCycler480^®^ 5x probe mastermix, 0.5 μL of each primer mix and 2 μL of each cDNA, 0.4 μl Universal ProbeLibrary (UPL) 10 μM and 7.1 μl nuclease free water. In each plate qPCR was performed with a cycle of 5 min 95°C, 55 cycles at 15s 95°C, 30s 60°C and 1s 72°C, to confirm amplification of specific transcripts. SP primers and corresponding UPL probe was performed using the ProbeFinder^™^ software (Version 2.04, Roche). To standardize mRNA concentration, the transcript levels of the housekeeping gene small ribosomal subunit (18S rRNA) were determined in parallel for each sample, and relative transcript levels were corrected by normalization based on the 18SrRNA transcript levels. The primers for 18S rRNA were as follows: forward 5′-GGT GCA TGG CCG TTC TTA-3′ and reverse 5′-TGC CAG AGT CTC GTT TA-3′. All real-time RT-PCR analyses were performed in triplicate, and the changes in gene expression were calculated by the ^ΔΔ^C_t_ method.

### Antibodies

The antibodies used were as follows: mouse anti-human surfactant protein A (SP-A) monoclonal antibody (MAB3270; Chemicon-Millipore, Hampshire, UK); mouse anti-human surfactant protein B (SP-B) monoclonal antibody (MAB3276; Chemicon-Millipore, Billerica, USA); rabbit anti-human SP-C (proSP-C) monoclonal antibody (AB3786; Chemicon-Millipore, Billerica, USA); rabbit anti-human SP-D monoclonal antibody (BM4083; Acris, San Diego, USA). Mouse*/Rabbit** anti-human Vimentin antibody (*M7020; DAKO—Glostrup, Denmark/** ab45939; Abcam) was used to characterize cells of Sertoli and Leydig. Secondary antibodies: anti-rabbit*/anti-mouse** IgG, respectively, conjugated to horseradish peroxidase (*P0448; Dako, Glostrup, Denmark/**80807; Dianova, Hamburg, Germany), anti-rabbit*/anti-mouse** IgG, respectively, conjugated to Alexa-Fluor 488 and Rhodamin-Red (*31665, **A11001, Thermo Fisher Scientific/life technologies). All antibodies mentioned were used for Western blot analysis as well as for immunohistochemical investigation as specified by the manufacturer. The antibodies used are highly specific, showing no cross-reactivity with other cellular proteins, and have been used successfully in previous experiments [11,12,16].

### Western blot analysis

For Western blots, testis and seminoma samples (standardized ratio: 100 mg wet weight/400 μm buffer containing 1% SDS and 4% 2-mercaptoethanol) were extracted as previously described in detail by Bräuer et al. [12]. The protein content of these samples, and of the collected seminal fluid samples, was measured with a protein assay based on the Bradford dye-binding procedure (BioRad, Hercules, CA). Total protein (100 μg) was then analyzed by Western blot. Proteins were resolved by reducing 15% SDS-polyacrylamide gel electrophoresis, electrophoretically transferred at room temperature for 1 h at 0.8 mA/cm^2^ onto 0.1 μm pore size nitrocellulose membranes and fixed with 0.2% glutaraldehyde in phosphate-buffered saline for 30 min. Bands were detected with primary antibodies to SP-A (1:500), SP-B (1:500), SP-C (1:500), SP-D (1:500, 0,5μg/μl) and secondary antibodies (anti-rabbit/anti-mouse IgG, respectively, conjugated to horseradish peroxidase, 1:5000) using chemiluminescence (ECL-Plus; Amersham-Pharmacia, Uppsala, Sweden). Human lung was used as the control. The molecular weights of the detected protein bands were estimated using standard proteins (Prestained Protein Ladder, Fermentas, St. Leon-Rot, Germany) ranging from 10 to 170 kDa.

### Immunohistochemistry

For immunohistochemistry, tissue specimens from healthy and pathological testicular tissue were embedded in paraffin, sectioned (6 μm) and dewaxed. Immunohistochemical staining was performed using antibodies to SP-A (1:50), SP-B (1:50), SP-C (1:50) and SP-D (1:50, 0,5μg/μl) as well as vimentin (1:100, 1 μg/ml). Antigen retrieval was performed by microwave pretreatment for 10 min and non-specific binding inhibited by incubation with normal serum (Dako) 1:5 in Tris-buffered saline (TBS). Each primary antibody was applied overnight at room temperature. The secondary antibodies (1:1000) were incubated at room temperature for at least 4 h. Visualization was achieved with diaminobenzidine (DAB) for at least 5 min. After counterstaining with hemalm, the sections were mounted in Aquatex (Boehringer, Mannheim, Germany). For immunofluorescence the secondary antibodies, respective fluorescein conjugated antibodies (Alexa 488, green and Rhodamin, red) diluted 1:1000 with TBS were used. Two negative control sections were used in each case: one was incubated with the secondary antibody only, and the other with the primary antibody only. Sections of human lung were used as the positive control. The slides were examined with a Keyence Biozero BZ81000E microscope.

### Enzyme-Linked Immunosorbent Assay (ELISA)

The ELISA-kits are from the company ELISAs USCN Life Science Inc. Wuhan. The analysis is performed using a microplate spectrophotometer (ELISA reader) at a wavelength of 450 nm, and 405 nm for measuring the absorbance. By comparing with the standard series and the determined values for antigen concentration (protein concentration), each sample can be calculated in ng / mg. The detection range for SP-A is 46.88-3000pg/ml, SP-B 1.56-100ng/ml, SP-C 0.313-20ng/ml and SP-D 6.25-400ng/ml.

### Statistical analysis

After evaluating the ELISA measurements with normal distribution using the Kolmogorov–Smirnov test, we derived one-way ANOVA statistics. For the interpretation of the results we used the Games-Howell post-hoc test because Levene's test for homogeneity of variances revealed an inequality of the variances.

All data were analyzed with the IBM SPSS Statistics software package version 19.0 (IBM SPSS, Chicago, IL). A p value less than 0.05 was considered statistically significant.

## Results

### Expression of surfactant proteins at mRNA level

**Fig 1:**
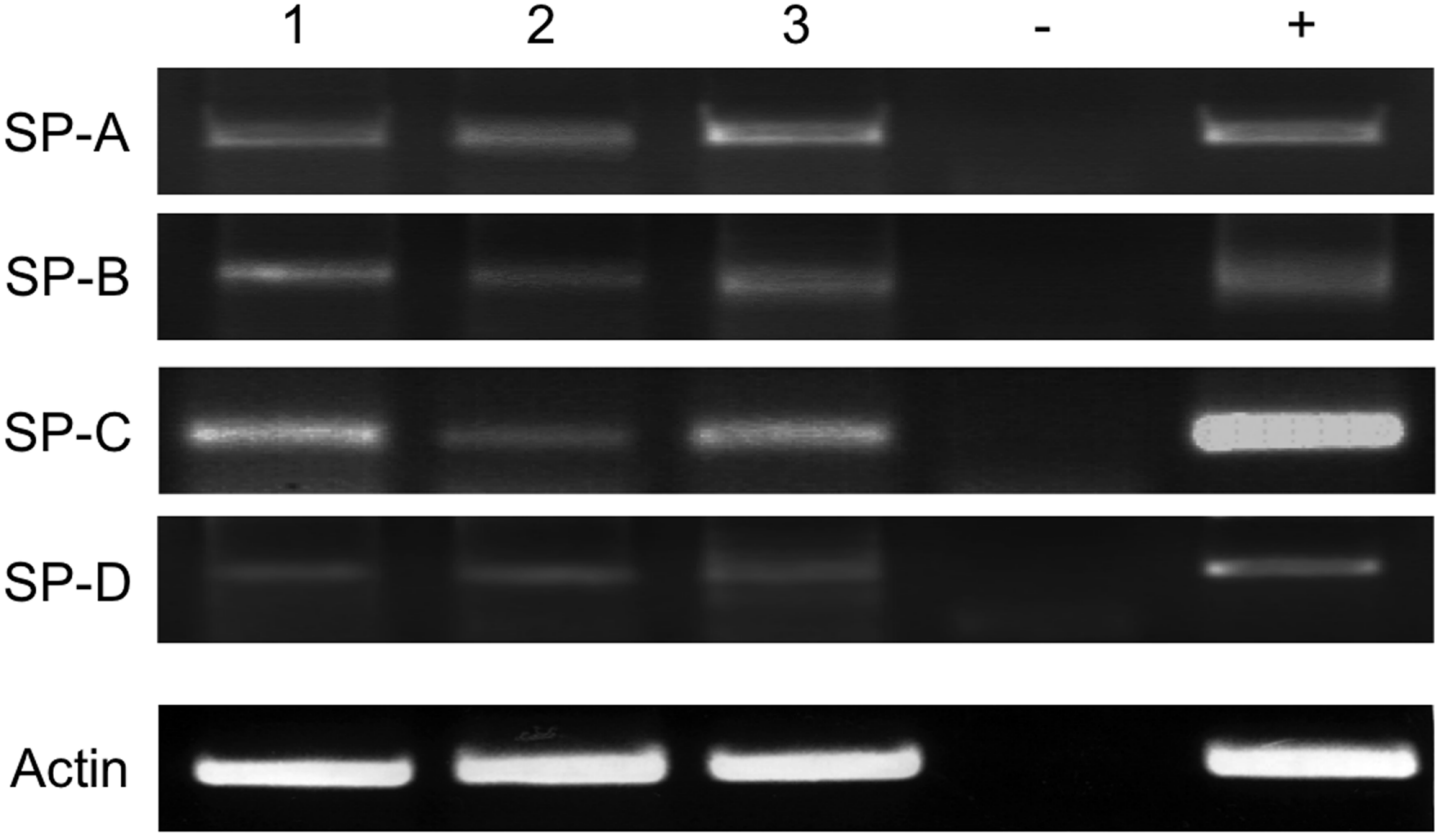
Visualization of RT-PCR amplification products from the following samples: tissue from different patients, (1) healthy testis 1; (2) healthy testis 2; (3) lung; (-) negative control (water); (+) positive control, plasmid DNA carrying the full-length gene for the respective surfactant proteins. All RT-PCR analyses show cDNA amplification for the relevant surfactant protein in comparison to ß-actin (ß-actin).

With primers specific for human surfactant proteins A, B, C, D (SP-A, SP-B, SP-C, SP-D), PCR products of the expected sizes, 212, 239, 110 and 139 bp, occurred in human healthy testis (Fig 1). Human lung also expressed these surfactant proteins. DNA bands for SPs in healthy testis and lung were compared to actin and reveal similar results. For verification, plasmids containing the open reading frame for the corresponding surfactant protein were used as positive control. Real-time RT-PCR quantification (qRT-PCR) revealed no significant alterations of SP-A, SP-B, SP-C and SP-D mRNA within healthy testis compared to peritumoral and/or tumoral testis (cf. S1 Fig).

### Expression of surfactant proteins at protein level

**Fig 2:**
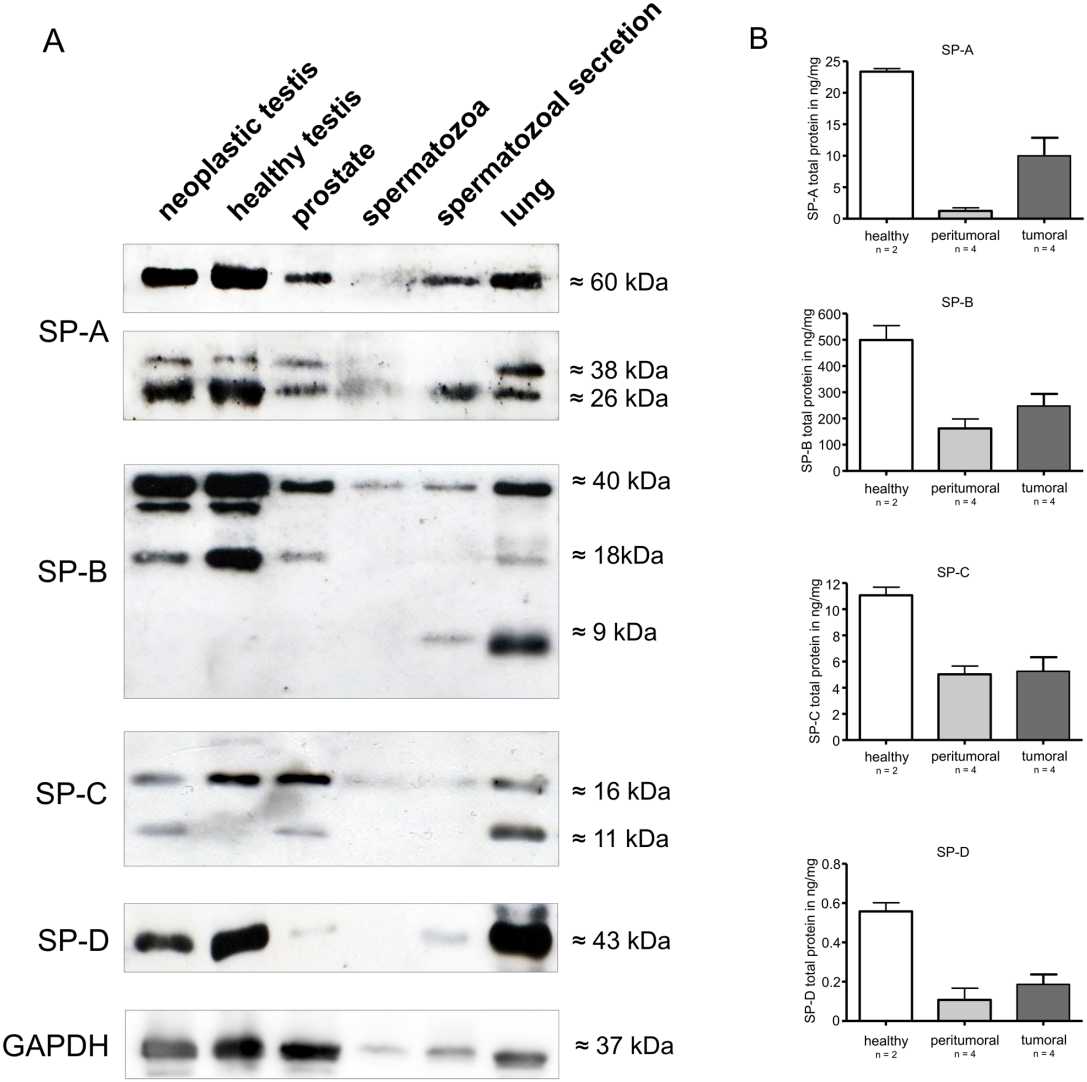
**A)** Western immunoblots of surfactant proteins A (SP-A), B (SP-B), C (SP-C) and D (SP-D) derived from the following samples: tissue from different patients (1) neoplastic testis; (2) healthy testis; (3) prostate; (4) spermatozoa (pellet); (5) spermatozoal secretion (supernatant); (6) lung. The proteins were separated by SDS-PAGE under reducing conditions and show distinct bands for all four investigated surfactant proteins at the specific molecular weights (SP-A: 60kDa, 38 kDa, 26 kDa; SP-B: 40, kDa, 18kDa, 9 kDa; SP-C: 16 kDa 11kDa; SP-D: 43 kDa). GAPDH was used as loading control at 37kDa. **B)** ELISA of SP-A, SP-B, SP-C and SP-D derived from the following samples: tissue from different patients, with healthy testis (n = 2) and with neoplastic (tumoral (n = 4) and peritumoral (n = 4)) testis. The protein concentration is expressed in ng/mg.

Western blots for surfactant proteins A, B, C, D revealed that human healthy testis expressed SP-A of the expected weights of ~60 kDa, ~38 kDa and ~26 kDa, SP-B of ~40kDa, ~18 kDa and ~9 kDa, SP-C of ~16 kDa and ~11kDa and SP-D of ~43 kDa (Fig 2). Compared to healthy testis, human seminoma expressed all surfactant proteins, especially SP-C, just ~16 kDa (Fig 2). Surfactant proteins A, B, C, D are all expressed in prostate. Surfactant proteins A, B, C and D were also detected in the supernatant of spermatozoal secretion, but not in spermatozoa. The expression pattern of surfactant proteins in human lung was similar to healthy human testis. However, in SP-B, beside the ~40 kDa and ~18 kDa proteins, a third band for a ~9 kDa protein was observed (Fig 2). Furthermore, at least weak protein bands could be detected in prostate for SP-A (~60 kDa, ~38 kDa and ~26 kDa) SP-B (~40kDa) and SP-C (~16 kDa).

### ELISA-Quantification of SP’s in human testis

ELISA quantification revealed higher concentrations of SP-A, SP-B, SP-C and SP-D within healthy testis when compared with peritumoral and tumoral (Fig 2B). The peritumoral and tumoral samples show in all samples a lower (but not significant) concentration compared to healthy testis. In Table 1 the mean values of the protein concentration is specified (cf. Table 1).

**Table 1:** ELISA Protein concentration of Surfactant proteins in ng/mg total protein.

Protein concentration mean value [ng / mg total protein]				
Surfactant Proteins	SP-A	SP-B	SP-C	SP-D
healthy testis	23.37	499.59	11.07	0.56
peritumoral testis	1.25	162.11	5.03	0.11
tumoral testis	9.98	247.98	5.26	0.19

### Immunohistochemistry of surfactant proteins

**Fig 3:**
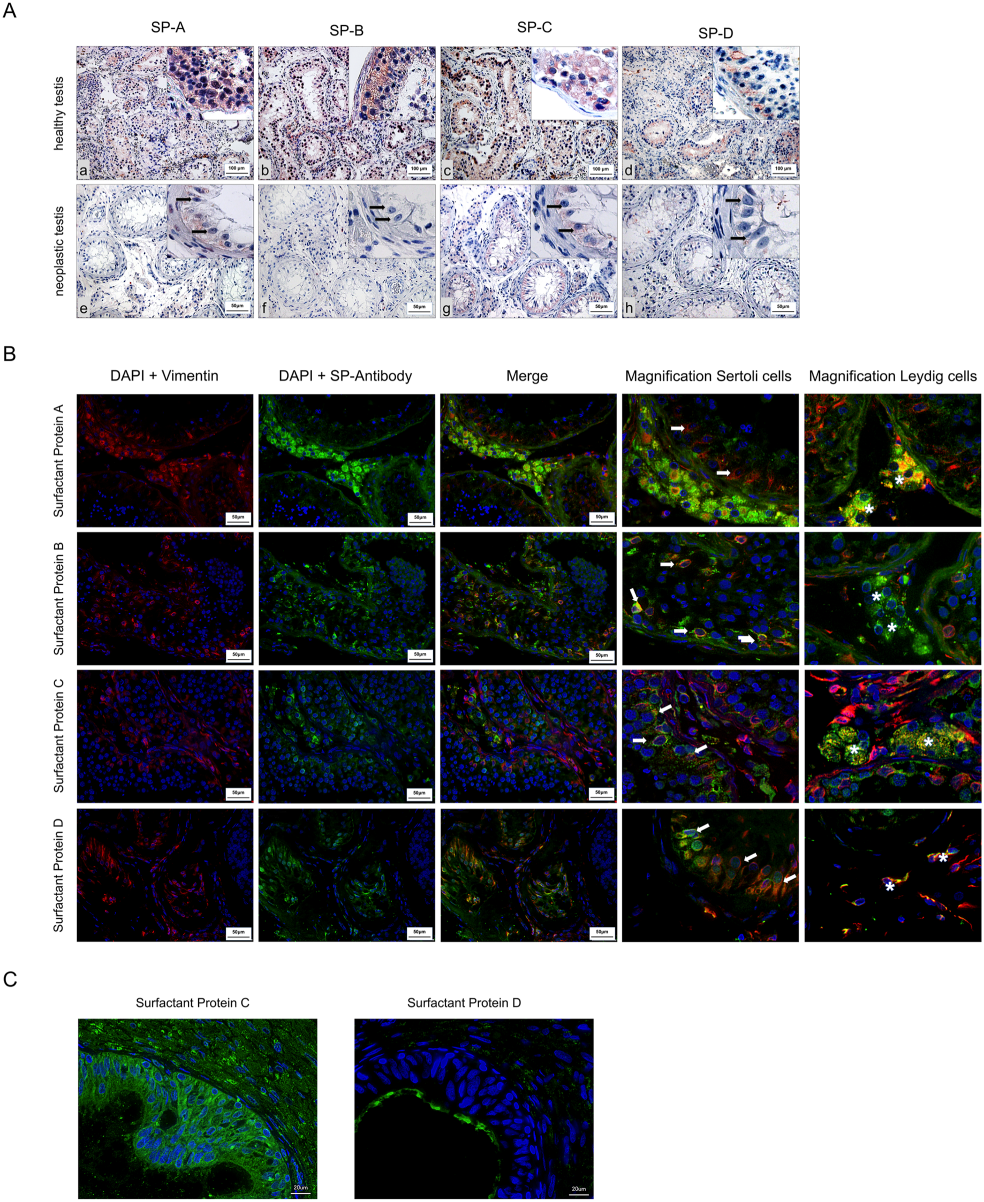
**A)** Immunohistochemical detection of surfactant proteins within healthy testis and neoplastic testis. In healthy testis, cells of seminiferous tubuli and cells of Leydig show weak immunostaining for SP-A and SP-D (a, d) and strong immunostaining for SP-B and SP-C (b, c). In Neoplastic testis, cells of seminiferous tubuli and cells of Sertoli (black arrow) show weak immunostaining for SP-A and SP-D (e, h). Strong immunostaining is obtained for SP-C while cells do not react with antibodies to SP-B (f, g). Scale bars: 50 μm. **B)** Fluorescence double-staining with Vimentin and surfactant protein. Cells of Sertoli (white arrow) show no double signal for SP-A, co-expression (yellow) with cells of Sertoli show SP-B, C and D, positive double-staining (yellow) for SP-A, B, C and D in cells of Leydig (white stars) **C)** Immunohistochemical detection of surfactant protein C and D in cells of the epididymal duct. Strong immunostaining (green) is obtained for SP-C in cytoplasm and for SP-D as a superficial layer.

In healthy testis, cytoplasm of all cells in seminiferous tubules reacted with antibodies to surfactant proteins A—D (Fig 3A). In detail, spermatogonia, cells of Sertoli, spermatocytes 1 and 2 were positive for the examined surfactant proteins. Furthermore, cells of Leydig showed a positive immunoreaction.

Compared with healthy testis, in seminoma, cytoplasm of all cells in seminiferous tubules and cells of Leydig (black arrow) revealed weakly or no reactivity to the used antibodies against surfactant proteins A, B, C and D (Fig 3A).

Cells of epididymal duct were immunostained by antibodies to surfactant proteins C and D (Fig 3C), but did not react with antibodies against surfactant proteins A and B (data not shown).

### Double fluorescence staining of cells of Sertoli and Leydig with vimentin

Cells of Sertoli and Leydig were identified using an antibody against vimentin, (Fig 3B, white arrow). The fluorescence analysis showed positive double signals (yellow) for SP-B, -C and -D within cells of Sertoli. SP-A is not located in cells of Sertoli. The analysis of cells of Leydig (Fig 3B, white stars) shows positive double signal (yellow) for all SP’s.

## Discussion

All results of this study show that surfactant proteins (SP-A, SP-B, SP-C and SP-D) are expressed in healthy human testis.

The mRNA of the surfactant proteins (A, B, C, D) was confirmed in all samples tested by RT-PCR analyses. The Western blot analyses revealed antibody reactivity and thus distinct protein bands at the expected molecular weights (SP-A: ~60 kDa, ~26 kDa and ~30 kDa, SP-B of ~40kDa and ~18 kDa, SP-C of ~16 kDa and ~11kDa and SP-D of ~43 kDa). In the case of SP-C, unprocessed (precursor) forms (pro-SPs) were detected in samples of healthy testis and seminoma with ~16 kDa. This fact has already been described for other tissue and results from intensive post-translational processing of the surfactant proteins [20,21]. As the presence of surfactant proteins within human and murine prostate has qualitatively already been described by others the corresponding results of this study are not entirely novel [22–24]. As the intensity of the protein bands obtained in the Western blot experiments does not stoichiometric correlate with the protein concentration only subjective quantification would be possible. For objective quantification of the surfactant protein concentrations we performed ELISA experiments. The mRNA expression was analyzed by means of real-time RT-PCR.

The immunohistochemical studies revealed readily distinguishable cytoplasmic distribution of the four investigated surfactant proteins SP-A, SP-B, SP-C and SP-D, mainly in spermatogonia, spermatocytes 1 and 2, cells of Sertoli and Leydig. Spermatocytes 2 differentiate into spermatids. On the way to becoming spermatozoa, spermatids turn their Golgi apparatus into an acrosome [25–27]. Functions attributed to cells of Sertoli are: secretion of androgen binding protein, inhibin, vimentin and delivery of a fluid for the transport of spermatozoa [26,27]. It can be assumed that SP-B and SP-C secreted by cells of Sertoli support the transport of spermatozoa in the direction of the rete testis. The dual nature of cells of Leydig comprises endocrine and neuroendocrine functions [28]. Their main function is the synthesis and secretion of testosterone. It can be assumed that SP-B and SP-C could support merocrine secretion of testosterone.

Either weak antibody reactivity or none at all was found in connective tissue. A comparable result in terms of the distribution of the surfactant proteins was already demonstrated in the epithelium of human nasal and oral mucosa and in the tear duct system [13,29].

The results of the present investigations reveal that surfactant proteins A and D seem to be inherent part of human testis. In this context and compared to previous findings we hypothesize that both proteins could also be components of the immune system of the male reproductive system, thus assigning to them a significant role in defense against pathogens within the environmentally exposed urogenital tract, like described in kidney and female genital tract [30,31]. In addition, rheological properties of SP-B and SP-C may be involved in the blood-testis-barrier [32]. This would be in accordance with a similar distribution of these proteins at the blood-cerebrospinal fluid barrier [33]. SP-A and SP-D also play important roles in cases of bacterial, viral and fungiform infections of the lungs. Levine (2001) showed that SP-D and SP-A are active in the modulation of cytokine production and inflammatory responses in bacterial pneumonia (e.g. *Streptococcus pneumoniae*) [34]. In addition, Ooi et al. and Schlosser et al. detected mRNA of SP-A and SP-D in healthy nasal mucosa with Chronic Rhinosinusitis (CRS), thus demonstrating the significance of surfactant proteins for mucociliary clearance and for inflammatory immunological processes [35,36].

In addition, the presence of SP-B and SP-C in the cells of seminiferous tubules and in the epididymal duct may influence the rheology of the seminal fluid, thus facilitating the clearance of the secretion. Efferent ducts contain cells with microvilli and ciliated cells [27]—surfactant proteins B and C may attenuate the function of these cells. The same fact can be attributed to the epididymal duct since its cells possess stereocilia involved in secretory and resorptive processes. A comparable rheological effect was observed in the epithelium of the nasal mucosa and Eustachian tube [37]. The presence of the surface proteins was demonstrated equally by several methods in all pathological tissue samples (PCR, Western blot, immunohistochemistry). Quantitative ELISA analysis of the surfactant proteins revealed decreased expression of surfactant proteins A, B, C, and especially D, in cases of seminoma. According to Holstein and Lauke (1996), tumor cells could influence other tumor cells in adjacent seminiferous tubules through a common microenvironment, for instance by growth factors [38]. It can be hypothesized that tumor cells may also influence normal testis cells, resulting in a decreased expression of surfactant proteins. Furthermore, it should be noted that cells of Sertoli in cases of seminoma differ from normal ones in that they contain glycogen and microfilaments [25] and therefore alter the SP expression. The additionally performed qRT-PCR revealed slight but not significant alterations with regard to the mRNA levels within the different samples. The fact that DNA/RNA levels do not necessarily correspond to the final protein concentration is not astonishing and also well described in variety of publications [39–41]. At last the mature protein defines function and activity.

It was shown that inflammatory processes correlate with increased surfactant protein expression, e.g. within the framework of inflammatory changes in human cornea [11] or in human prostate [42]. Nasal mucosa of patients with chronic rhinosinusitis with polyps, on the other hand, showed no change in protein concentration of SPs [43]. The reduction in SPs reported here might reflect an impairment of testis function.

The presence of surfactant protein A, B, C and D in healthy testis was demonstrated for the first time in this study. The fact that SP-C is an important factor in stabilization of the biofilms at air-fluid interfaces has already been demonstrated in numerous studies [44,45]. On the other hand, no studies to date have discerned a connection between expression rate of SP-C in cells of seminiferous tubules or ductus epididymis and the rheological properties of the seminal fluid. In epithelial cells of the nasal mucosa, an intact fluid film and mucociliary transport are important factors in the self-cleaning and natural defense function of the mucosa. This important function involves the surfactant proteins and the surfactant itself [46]. Dysregulation of the surfactant system, e.g. in various forms of rhinitis, results in negative changes of the moisture balance as well as the consistency of the mucus. It is conceivable that impairment of surfactant proteins is due to rheological and immunodefensive disorders of testis and epididymis or prostate.

## Supporting Information

S1 Fig Quantitative Real-Time RT-PCR analysis of Surfactant Protein A, B, C and D. The fold increase transcript levels are shown as mean ± SEM and statistical significance vs. healthy testis (no significance).
(TIF)
