# Correction: Expression and Localization of Lung Surfactant Proteins in Human Testis

**DOI:** 10.1371/journal.pone.0317060

**Published:** 2025-01-03

**Authors:** Stephanie Beileke, Horst Claassen, Walter Wagner, Cord Matthies, Christian Ruf, Arndt Hartmann, Fabian Garreis, Friedrich Paulsen, Martin Schicht, Lars Bräuer

The following information is missing from the Acknowledgements section: This work was carried out in fulfillment of the requirements for the degree of Dr. med. (SB).
