# Supplementary figures and images for: Expression and Localization of Lung Surfactant Proteins in Human Testis
Source: PLoS One. 2015 Nov 24;10(11):e0143058. doi: 10.1371/journal.pone.0143058 (PMC4658200; doi:10.1371/journal.pone.0143058)

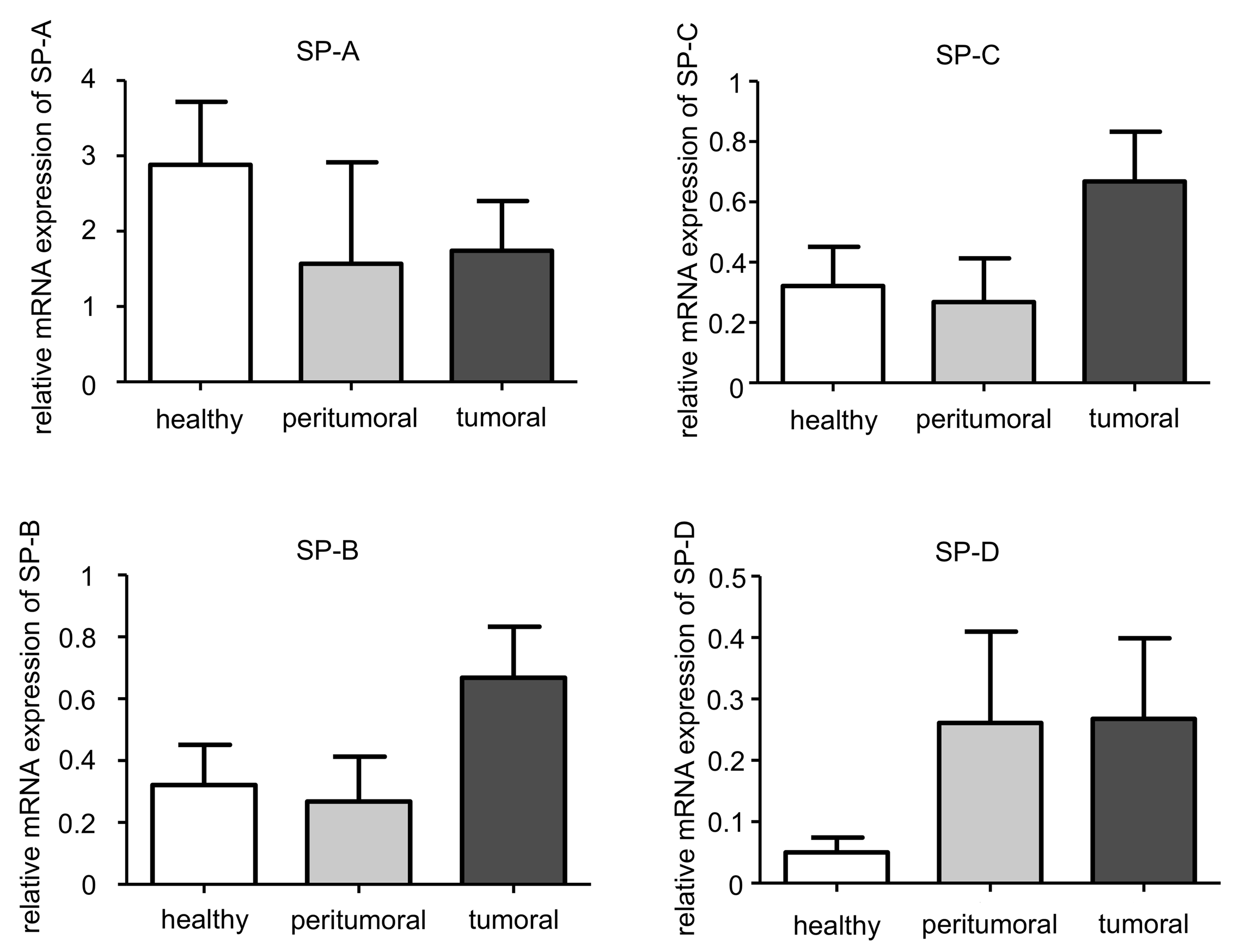

Supplement: S1 Fig — The fold increase transcript levels are shown as mean ± SEM and statistical significance vs. healthy testis (no significance). (TIF) [file pone.0143058.s001.tif]
